# The Impact of Frailty on Postoperative Cardiopulmonary Complications in the Emergency General Surgery Population

**DOI:** 10.1055/s-0038-1655756

**Published:** 2018-05-23

**Authors:** Serra Akyar, Sarah J. Armenia, Parita Ratnani, Aziz M. Merchant

**Affiliations:** 1 Department of Surgery, New Jersey Medical School, Rutgers Biomedical and Health Sciences, Rutgers University, Newark, New Jersey

**Keywords:** emergency general surgery, frailty, postoperative complications, surgical outcomes, risk factors

## Abstract

**Background** The burden of frail patients undergoing emergency general surgery (EGS) is increasing rapidly and this population is particularly susceptible to postoperative cardiopulmonary complications and mortality. We aimed to determine the association between frailty, as defined by the previously described modified frailty index (mFI), and postoperative respiratory complications (unplanned reintubation, pneumonia, and prolonged ventilation), cardiac complications (myocardial infarction and cardiac arrest), and mortality. We also sought to identify the most significant determinants of frailty in the highest risk patients based on the specific variables comprising the mFI.

**Methods** We performed a retrospective observational analysis of the prospectively collected American College of Surgeons National Surgical Quality Improvement Program database. Files from 2005 to 2015 identified 132,765 inpatients who underwent EGS. mFI scores were calculated for each patient. The effect of increasing frailty on unplanned reintubation, pneumonia, prolonged ventilation, myocardial infarction, cardiac arrest, and mortality was evaluated using bivariate analysis. Multivariable logistic regression was used to compare mFI with additional predictor variables including race, gender, physical status as defined by the American Society of Anesthesiologists, disseminated cancer, renal failure, smoking status, sepsis, wound presence/classification, dyspnea, and previous ventilator dependence.

**Results** Unplanned reintubation, pneumonia, prolonged ventilation, myocardial infarction, cardiac arrest, and mortality were significantly associated with frailty, and the odds of each postoperative complication increased with increasing mFI score. Of the frailest patients (mFI ≥3) that experienced cardiopulmonary complications or mortality, the variables of the mFI that contributed most to frailty were hypertension requiring medication and functional status before surgery.

**Conclusions** A higher mFI score is associated with increased odds of postoperative cardiopulmonary complications and mortality in the EGS population. Specific variables of the mFI can also provide valuable information for assessing odds in the frailest patients undergoing EGS.

The United States Census Bureau estimates that the number of people > 65 years of age will double between 2010 and 2050. 1 Patients > 65 years old currently account for ∼50% of all emergent operations and 75% of operative mortality. 2 The expansion of this demographic is correlated with an increasing prevalence of frail patients that may present for emergency general surgery (EGS). 3 Frailty, rather than chronologic age, is becoming increasingly recognized as a proxy for physiological reserve in the elderly and an important predictor of surgical outcomes. 4 It is an independent risk factor for postoperative morbidity, increased resource use, and mortality. 5 Postoperative complications are more likely to result in mortality in the frail population, and are associated with increased length of stay and hospital costs. 6 The burden of frail patients undergoing EGS is increasing rapidly; however, there are still few studies exploring the relationship between frailty and postoperative complications in the EGS population.

Cardiopulmonary complications are the principal causes of death in elderly patients following bowel resection, comprising 55% of all deaths in one study. 7 Elderly patients undergoing gastrointestinal surgery are between 14 and 18 times more likely to experience cardiac complications (such as cardiac arrest) and between 1.5 and 5.1 times more likely to experience pulmonary complications (such as pneumonia and unplanned reintubation) than younger patients. 8 These patients also experience greater morbidity associated with cardiac or pulmonary postoperative complications. 7 9 10 Several studies assessing the burden of specific complications in the EGS setting have identified that reducing the incidence of postoperative pneumonia offers the greatest value. 9 11 12 Given the significant burden of cardiopulmonary complications in the elderly population and in the EGS setting as a whole, further study of cardiopulmonary complications in frail patients undergoing EGS is warranted.

To our knowledge, there are no studies exploring the association of frailty and specific cardiac and respiratory complications in the EGS population. In this study, we aimed to determine the association between frailty and postoperative respiratory complications (unplanned reintubation, pneumonia and prolonged ventilation), cardiac complications (myocardial infarction and cardiac arrest), and mortality. We also aimed to identify the most significant determinants of frailty in the highest risk patients based on specific modified frailty index (mFI) variables.

## Methods

### Data Source

We performed a retrospective observational analysis of the American College of Surgeons National Surgical Quality Improvement Program (NSQIP) database between the years 2005 and 2015. This study was approved by the Institutional Review Board of New Jersey Medical School. The NSQIP database is a multicenter database of prospectively collected data. It was created to facilitate quality improvement in hospital surgical care by collecting clinical data consisting of demographic variables, preoperative risk factors, intraoperative information, and 30-day postoperative outcomes. 13 Data are deidentified and collected by trained abstractors to ensure robust and high-quality data entry. 14 As a validated and comprehensive program with risk-adjusted surgical outcomes, the NSQIP database has shown to be an effective source for data in observational studies investigating clinical outcomes.

### Study Population

Our study included adult patients who underwent EGS in the NSQIP database between 2005 and 2015. To identify our study population, we selected patients who underwent general surgery cases. We then limited the study to emergency and nonelective cases. Next, we extracted only those cases that matched Gale et al's 15 definition of EGS. Gale et al identified International Classification of Disease—9th Revision (ICD-9) codes that correspond with EGS-defining diagnoses as identified by the American Association for the Surgery of Trauma. 16 Since the NSQIP database did not contain ICD-9 codes for every year of our study, we matched the ICD-9 codes identified by Gale et al to their corresponding current procedural terminology (CPT) codes. Using the identified CPT codes, we extracted EGS specific cases from the NSQIP database. Appendix A provides the final list of CPT codes used to identify EGS procedures for inclusion in this study.

**Appendix A:** Current procedural terminology codes used to identify open and laparoscopic emergency general surgery procedures

Colectomy	Enterectomy	Cholecystectomy	Gastrorrhaphy	Adhesiolysis	Appendectomy
44,141	44,120	47,600	43,840	44,005	44,950
44,144	44,121	47,605	44,602	44,180	44,955
44,145	44,125	47,610	44,603		44,960
44,146	44,110	47,612	43,501		44,970
44,147	44,111	47,562	43,502		
44,173	44,202	47,563			
44,155	44,203	47,564			
44,156					
44,160					
44,139					
44,140					
44,205					
44,207					
44,208					
45,395					
45,397					
44,204					
44,206					
44,212					
44,213					

### Modified Frailty Index

mFI scores were used to assess frailty. Saxton and Velanovich 17 first described mFI by matching 11 of the 70 variables from the Canadian Study of Health and Aging Frailty Index to 15 variables found in the NSQIP dataset. The 15 NSQIP variables can be found in Appendix B . mFI has been validated for use in the EGS population using NSQIP. 18 A patient's mFI is calculated as the number of variables present in a patient's medical history from the total 11 variables. 19 Patients with mFI scores ≥4 were grouped together to prevent variability associated with small group sizes. Patients without mFI variables were scored as 0 and included in the analysis. Patients with missing information for any of the variables listed in Appendix B were excluded.

**Appendix B:** National Surgical Quality Improvement Program (NSQIP) variables used to generate modified frailty index variable

NSQIP variable	Description
CPNEUMON	Current pneumonia
CVA	CVA/Stroke with neurological deficit
CVANO	CVA/Stroke with no neurological deficit
FNSTATUS2	Functional health status prior to surgery
HXANGINA	History of angina 1 month before surgery
HXCHF	Congestive heart failure 30 days before surgery
HXCOPD	History of severe COPD
HXDM	Diabetes
HXMI	History of myocardial infarction 6 months before surgery
HXPVD	History of revascularization/amputation for peripheral vascular disease
HXTIA	History of transient ischemic attacks
HYPERMED	Hypertension requiring medication
IMPSENS	Impaired sensorium
PRVPCI	Previous percutaneous coronary intervention
PRVPCS	Previous cardiac surgery

**Abbreviations:** COPD, chronic obstructive pulmonary disease; CVA, cerebrovascular accident.

### Predictor Variables

Additional predictor variables were also tested, including wound infection, disseminated cancer, renal failure, wound classification, smoking status, American Society of Anesthesiologists (ASA) classification, sepsis, race, gender, dyspnea, ventilator dependence before surgery, and length of operation. These variables were identified in previous literature as potential predictor variables. 20 We studied the relationship between these variables, mFI, and the outcomes.

### Outcome of Interest

Our study had six primary outcomes: occurrence of unplanned intubation following surgery, occurrence of pneumonia following surgery, occurrence of prolonged ventilation (defined as use of a ventilator for >48 hours following surgery), occurrence of cardiac arrest within 30 days following surgery, occurrence of myocardial infarction within 30 days following surgery, and death within 30 days following surgery. Each outcome was dichotomized and studied separately with the main predictor (mFI), and with additional predictor variables.

### Statistical Analysis

Univariate and bivariate analyses were used to study mFI, the six outcomes, and additional predictor variables. Six separate multivariable logistic regression analyses were then performed, one for each primary outcome. A stepwise multivariable analysis with backward elimination was used to determine the best-fit model, with mFI acting as the main predictor. Hosmer–Lemeshow tests, C-statistics, and sensitivity analyses were performed, and variance inflation factors (VIF) were derived. These analyses helped establish goodness-of-fit and determine the presence or absence of multicollinearity.

To study the most common determinants of frailty, mFI ≥ 3 were used to form a subgroup of the frailest patients from the total study population. The six initial outcomes were regrouped into larger categories: all respiratory adverse outcomes (occurrence of unplanned intubation following surgery, occurrence of pneumonia following surgery, and occurrence of prolonged ventilation defined as use of a ventilator >48 hours following surgery) and all cardiac adverse outcomes (occurrence of cardiac arrest within 30 days following surgery and occurrence of myocardial infarction within 30 days following surgery). Bivariate analyses were performed between each of the 15 variables listed in Appendix B and all respiratory adverse outcomes, all cardiac adverse outcomes, and mortality. If a patient had > 1 individual respiratory or cardiac adverse outcome within its larger group, that outcome was counted only once.

For all univariate, bivariate, and regression models, a *p* value < 0.05 was considered significant. Data were entered and analyzed using SAS software (SAS Institute, Cary, NC), version 9.4.

## Results

The study population included a total of 132,922 patients who underwent EGS between 2005 and 2015, inclusive. Due to missing data needed to tabulate the mFI score, 157 patients were excluded. A final total of 132,765 adult patients were included in our study ( Table 1 ). The majority of patients had mFI scores of 0, 1, or 2 (90.7%).

**Table 1:** Descriptive statistics of patient demographics

Variable	*N* (% of Total Sample)
**Age**	
< 40	54,039 (40.7%)
41–60	40,323 (30.37%)
61–80	28,654 (21.58%)
> 81	7,998 (6.02%)
Missing	1,751 (1.31%)
**Gender**	
Male	64,259 (48.40%)
Female	68,185 (51.35%)
Missing	321 (0.24%)
**Ethnicity**	
White	72,383 (54.51%)
Black	9,070 (6.83%)
Asian	3,953 (2.97%)
American Indian/Alaska Native	1,069 (0.80%)
Native Hawaiian/Pacific Islander	408 (0.30%)
Missing	45,882 (34.55%)
**mFI** a	
0	81,250 (61.20%)
1	25,555 (19.25%)
2	13,614 (10.25%)
3	6,959 (5.24%)
4+	5,387 (4.06%)
** ASA class b**	
1	30,271 (22.80%)
2	56,904 (42.86%)
3	29,627 (22.31%)
4	14,017 (10.55%)
5	1,764 (1.32%)
Missing	182 (0.06%)
**Smoking status**	
Yes	28,640 (21.57%)
**Acute renal failure**	
Yes	2,290 (1.72%)
**Dyspnea**	
At rest	4,037 (3.04%)
Moderate exertion	5,443 (4.10%)
No	12,3284 (92.86%)
Missing	1 (0%)
**Disseminated cancer**	
Yes	2,368 (1.78%)
**Wound infection**	
Yes	3,688 (2.78%)
**Prior operation within 30 days**	
Yes	5,959 (4.49%)
Missing	7,394 (5.57%)

a Modified frailty index.

b American Society of Anesthesiologists Classification.

### Patient Demographics

Patient demographics and comorbid conditions are shown in Table 1 . The distribution of mFI cohorts by patient age is shown in Table 2 . Of note, 28.8% of patients were > 60 years of age. Of those classified with an mFI score of 0, 61.04% were < 41 years. Conversely, those classified with mFI scores ≥ 3 had a greater proportion of patients > 60 years compared with those with mFI scores ≤ 2 (74.95 vs 22.75%, respectively).

**Table 2:** Distribution of modified frailty index cohorts by patient age

Age (% of total sample)	mFI				
	0	1	2	3	4+
< 40 (40.70%)	49,603	3,523	691	157	65
41–60 (30.37%)	24,044	9,990	3,907	1,474	908
61–80 (21.58%)	6,609	9,116	6,275	3,617	3,037
> 81 (6.02%)	824	2,372	2,202	1,400	1,200
Missing (1.31%)	170	554	539	311	177

Abbreviation: mFI, modified frailty index.

### Respiratory Outcomes

Table 3 presents results from the adjusted multivariable logistic regression models examining the association between frailty and unplanned reintubation, pneumonia, and prolonged ventilation. Tables 4 , 5 , and 6 show the results of Hosmer–Lemeshow testing and C-statistics, sensitivity analyses, and VIF testing, respectively. Patients with an mFI score of 1 experienced 82% greater odds of unplanned reintubation than patients with an mFI score of 0 (odds ratio [OR]: 1.826, 95% confidence interval [CI:] 1.611–2.070). The odds increased with increasing mFI score, and patients with mFI scores ≥ 4 were 2.93 times more likely to require an unplanned reintubation than patients with an mFI score of 0 (OR: 2.934, 95% CI: 2.534–3.398). The odds of having pneumonia also increased with increasing mFI score, with patients with an mFI score of 1 having a 66% greater odds of having pneumonia postoperatively compared with those with an mFI score of 0 (OR: 1.669, 95% CI: 1.494–1.864). Additionally, the odds of requiring prolonged ventilation increased with increasing mFI score; patients with mFI ≥4 had 2.5 (OR: 2.511, 95% CI: 2.255–2.796) times the odds of requiring prolonged ventilation compared with those with an mFI score of 0 and those with an mFI of 1 had 1.66 (OR: 1.667, 95% CI: 1.519–1.829) times the odds of requiring prolonged ventilation compared to those with an mFI score of 0.

**Table 3:** Adjusted odds ratios between frailty and postoperative cardiopulmonary complications and mortality in emergency general surgery

Respiratory complications						
	Unplanned reintubation a		Pneumonia b		Prolonged ventilation c	
mFI	OR (95% CI)	*p* Value	OR (95% CI)	*p* Value	OR (95% CI)	*p* Value
0	Ref		Ref		Ref	
1	1.826 (1.611–2.070)	<0.0001	1.669 (1.494–1.864)	<0.0001	1.667 (1.519–1.829)	<0.0001
2	2.387 (2.099–2.716)	<0.0001	2.074 (1.849–2.327)	<0.0001	2.059 (1.872–2.264)	<0.0001
3	2.877 (2.505–3.305)	<0.0001	2.190 (1.930–2.484)	<0.0001	2.386 (2.154–2.644)	<0.0001
4+	2.934 (2.534–3.398)	<0.0001	2.163 (1.892–2.472)	<0.0001	2.511 (2.255–2.796)	<0.0001
**Cardiac complications**						
	**Cardiac arrest** d		**Myocardial infarction** e			
**mFI**	**OR (95% CI)**	***p*** **Value**	**OR (95% CI)**	***p*** **Value**		
0	Ref		Ref			
1	1.815 (1.396–2.360)	<0.0001	9.862 (7.051–13.792)	<0.0001		
2	2.378 (1.828–3.094)	<0.0001	21.517 (15.463–29.942)	<0.0001		
3	2.861 (2.179–3.757)	<0.0001	32.805 (23.316–46.156)	<0.0001		
4+	3.359 (2.552–4.422)	<0.0001	41.794 (29.554–59.104)	<0.0001		
**Mortality** f						
**mFI**	**OR (95% CI)**	***p*** **Value**				
0	Ref					
1	1.949 (1.719–2.209)	<0.0001				
2	2.885 (2.547–3.276)	<0.0001				
3	3.504 (3.077–3.991)	<0.0001				
4+	4.459 (3.904–5.092)	<0.0001				

Abbreviations: ASA, American Society of Anesthesiologists; CI, confidence interval; mFI, modified frailty index; OR, odds ratio; Ref, reference category.

a Adjusted for ASA class, gender, operation time, sepsis, smoker status, ventilator dependence, wound infection.

b Adjusted for ASA class, dyspnea, gender, operation time, sepsis, smoker status, ventilator dependence, wound class, wound infection.

c Adjusted for ASA class, dyspnea, operation time, renal failure, sepsis, smoker status, ventilator dependence, wound class, wound infection.

d Adjusted for ASA class, dyspnea, gender, operation time, renal failure, sepsis, ventilator dependence.

e Adjusted for renal failure, sepsis, wound infection.

f Adjusted for ASA class, disseminated cancer, dyspnea, gender, renal failure, sepsis, smoker status, ventilator dependence, wound infection.

**Table 4:** Hosmer–Lemeshow test results and C-statistics

Outcomes	Hosmer–Lemeshow *p* Value	C-statistic
Unplanned reintubation a	< 0.0001	0.869
Pneumonia b	< 0.0001	0.864
Prolonged ventilation c	< 0.0001	0.934
Cardiac arrest d	< 0.0001	0.904
Myocardial infarction e	< 0.0001	0.836
Mortality f	< 0.0001	0.940

Abbreviation: ASA, American Society of Anesthesiologists.

a Adjusted for ASA class, gender, operation time, sepsis, smoker status, ventilator dependence, wound infection.

b Adjusted for ASA class, dyspnea, gender, operation time, sepsis, smoker status, ventilator dependence, wound class, wound infection.

c Adjusted for ASA class, dyspnea, operation time, renal failure, sepsis, smoker status, ventilator dependence, wound class, wound infection.

d Adjusted for ASA class, dyspnea, gender, operation time, renal failure, sepsis, ventilator dependence.

e Adjusted for renal failure, sepsis, wound infection.

f Adjusted for ASA class, disseminated cancer, dyspnea, gender, renal failure, sepsis, smoker status, ventilator dependence, wound infection.

**Table 5:** Sensitivity analysis: *p* values of unadjusted and adjusted multivariable logistic regression model variables

Respiratory complications						
	Unplanned reintubation		Pneumonia		Prolonged ventilation	
Predictor variables	Unadjusted	Adjusted a	Unadjusted	Adjusted a	Unadjusted	Adjusted a
ASA class	<0.0001	<0.0001	<0.0001	<0.0001	<.0001	<.0001
Disseminated cancer	0.1169	–	0.275	–	0.7042	–
Dyspnea	<0.0001	–	<0.0001	<0.0001	<.0001	<.0001
Gender	0.0015	<0.0001	<0.0001	<0.0001	0.5354	–
Modified frailty index b	<0.0001	<0.0001	<0.0001	<0.0001	<0.0001	<0.0001
Operation time	<0.0001	<0.0001	<0.0001	<0.0001	<0.0001	<0.0001
Renal failure	0.0035	–	0.2356	–	<0.0001	<0.0001
Sepsis	<0.0001	<0.0001	<0.0001	<0.0001	<0.0001	<0.0001
Smoker status	0.0021	<0.0001	<0.0001	<0.0001	<0.0001	<0.0001
Ventilator dependence	0.0289	0.0004	<0.0001	<0.0001	<0.0001	<0.0001
Wound classification	0.3333	–	0.0019	0.0019	0.0019	0.0009
Wound infection	0.0172	0.0022	<0.0001	<0.0001	<0.0001	<0.0001
**Cardiac complications and mortality**						
	**Cardiac arrest**		**Myocardial infarction**		**Mortality**	
**Predictor variables**	**Unadjusted**	**Adjusted** a	**Unadjusted**	**Adjusted** a	**Unadjusted**	**Adjusted** a
ASA class	<0.0001	0.005	0.9268	–	<0.0001	<0.0001
Disseminated cancer	0.8074	–	0.2267	–	<0.0001	<0.0001
Dyspnea	0.0444	0.0380	0.8665	–	<0.0001	<0.0001
Gender	0.0219	0.0220	0.3961	–	<0.0001	<0.0001
Modified frailty index b	<0.0001	<0.0001	<0.0001	<0.0001	<0.0001	<0.0001
Operation time	<0.0001	<0.0001	0.0561	–	0.4199	–
Renal failure	<0.0001	<0.0001	0.0137	0.0023	<0.0001	<0.0001
Sepsis	<0.0001	<0.0001	0.0108	<0.0001	<0.0001	<0.0001
Smoker status	0.4013	–	0.0932	–	<0.0001	<0.0001
Ventilator dependence	<0.0001	<0.0001	0.1152	–	<0.0001	<0.0001
Wound classification	0.3066	–	0.2325	–	0.3234	–
Wound infection	0.0899	–	0.0057	0.0156	0.0063	0.0025

Abbreviation: ASA class, American Society of Anesthesiologists classification of physical status.

a A “–” listed in the adjusted column for the outcome indicates that the corresponding variable was removed and not included in the final model.

b Modified frailty index is the main predictor of all models.

**Table 6:** Variance inflation factor test results

Respiratory complications			
	Unplanned reintubation a	Pneumonia b	Prolonged ventilation c
Predictor variables	Variance inflation	Variance inflation	Variance inflation
ASA class	2.068	2.069	2.070
Disseminated cancer	–	–	–
Dyspnea	1.225	1.225	1.228
Gender	1.007	1.011	–
Operation time	1.146	1.153	1.153
Renal failure	–	–	1.091
Sepsis	1.077	1.159	1.161
Smoker status	1.019	1.010	1.015
Ventilator dependence	1.256	1.257	1.281
Wound classification	–	1.095	1.091
Wound infection	1.107	1.108	1.108
**Cardiac complications and mortality**			
	**Cardiac arrest** d	**Myocardial infarction** e	**Mortality** f
**Predictor variables**	**Variance inflation**	**Variance inflation**	**Variance inflation**
ASA class	2.050	–	2.003
Disseminated cancer	–	–	1.028
Dyspnea	1.226	–	1.229
Gender	1.004	–	1.008
Operation time	1.145	–	–
Renal failure	1.092	1.055	1.092
Sepsis	1.077	1.052	1.079
Smoker status	–	–	1.019
Ventilator dependence	1.249	–	1.278
Wound classification	–	–	–
Wound infection	–	1.069	1.107

Abbreviation: ASA class, American Society of Anesthesiologists classification of physical status.

a Adjusted for ASA class, gender, operation time, sepsis, smoker status, ventilator dependence, wound infection.

b Adjusted for ASA class, dyspnea, gender, operation time, sepsis, smoker status, ventilator dependence, wound class, wound infection.

c Adjusted for ASA class, dyspnea, operation time, renal failure, sepsis, smoker status, ventilator dependence, wound class, wound infection.

d Adjusted for ASA class, dyspnea, gender, operation time, renal failure, sepsis, ventilator dependence.

e Adjusted for renal failure, sepsis, wound infection.

f Adjusted for ASA class, disseminated cancer, dyspnea, gender, renal failure, sepsis, smoker status, ventilator dependence, wound infection.

### Cardiac Outcomes

Table 3 presents results from the adjusted multivariable logistic regression models examining the association between frailty and cardiac arrest, and frailty and myocardial infarction. Tables 4 , 5 , and 6 show the results of Hosmer–Lemeshow testing and C-statistics, sensitivity analyses, and VIF testing, respectively. With increasing mFI score, the odds of both cardiac arrest and myocardial infarction increased when each mFI score category was compared with mFI scores of 0. Patients with an mFI of 1 experienced > 9 times the odds of myocardial infarction than patients with an mFI of 0 (OR: 9.862, 95% CI: 7.051–13.792). Patients with an mFI ≥4 had >41 times the odds of experiencing a myocardial infarction than patients with an mFI of 0 (OR: 41.794, 95% CI: 29.554–59.104).

### Mortality

Table 3 presents results from the adjusted multivariable logistic regression models examining the association between frailty and mortality. Tables 4 , 5 , and 6 show the results of Hosmer–Lemeshow testing and C-statistics, sensitivity analyses, and VIF testing, respectively. Patients with an mFI score of 1 experienced 94% higher odds of mortality than patients with an mFI of 0 (OR: 1.949, 95% CI: 1.719–2.209). The odds increased with increasing mFI scores, and patients with an mFI ≥4 had 4.4 times the odds of death than patients with an mFI of 0 (OR: 4.459, 95% CI: 3.904–5.092).

### Determinants of Frailty

A total of 12,346 patients had mFI scores ≥ 3 and comprised the subgroup of frailest patients used to identify the determinants of frailty. As described in the Methods section of this paper, the mFI score was created by compiling 15 specific NSQIP variables ( Appendix B ). Table 7 shows the frequency of these variables in the frailest patient subgroup who experienced postoperative respiratory complications (unplanned reintubation, pneumonia, or prolonged ventilation). Similarly, Table 8 shows this information for those of the frailest patients that experienced postoperative cardiac complications (myocardial infarction or cardiac arrest), while Table 9 shows this information for those of the frailest patients who died. Among the frailest patients who experienced either a respiratory complication, cardiac complication, or death, it was found that hypertension requiring medication was the most frequently encountered mFI variable. It contributed to the frailty of 33.17% of patients who experienced a respiratory complication, 5.97% of patients who experienced a cardiac complication, and 23.36% of patients who died. The next most impactful mFI variable contributing to cardiac complication, respiratory complications, and mortality, was the patient's functional health status prior to surgery (whether or not the patient was partially or completely dependent preoperatively). Functional health status prior to surgery contributed to the frailty of 29.47% of patients who experienced a respiratory complication, 4.98% of patients who experienced a cardiac complication, and 22.22% of patients who died.

**Table 7:** Descriptive statistics of individual modified frailty index variables (mFI) in the frailest population (patients with mFI ≥ 3) that experienced respiratory complications

mFI variable	Respiratory outcome	
	Yes (%)	No (%)
Hypertension requiring medication	33.17%	4.87%
Functional health status prior to surgery	29.47%	8.57%
Diabetes	15.66%	22.37%
CVA/Stroke with neurological deficit	13.12%	86.88%
History of severe COPD	11.98%	26.06%
Impaired sensorium	10.87%	27.17%
Previous cardiac surgery	9.21%	28.83%
Current pneumonia	7.33%	30.71%
Previous percutaneous coronary intervention	6.93%	31.1%
Congestive heart failure 30 days before surgery	6.73%	31.31%
History of myocardial infarction 6 months before surgery	4.13%	33.9%
History of revascularization/amputation for peripheral artery disease	3.8%	34.24%
CVA/Stroke with no neurological deficit	3.40%	34.64%
History of transient ischemic attacks	2.95%	35.08%
History of angina 1 month before surgery	2.00%	36.04%

Abbreviations: COPD, chronic obstructive pulmonary disease; CVA, cerebrovascular accident; mFI, modified frailty index.

**Table 8:** Descriptive statistics of individual modified frailty index variables (mFI) in the frailest population (patients with mFI ≥ 3) that experienced cardiac complications

mFI variable	Cardiac outcome	
	Yes (%)	No (%)
Hypertension requiring medication	5.97%	0.79%
Functional health status prior to surgery	4.98%	1.79%
Diabetes	2.96%	3.80%
History of severe COPD	2.11%	4.65%
Previous cardiac surgery	1.92%	4.84%
Impaired sensorium	1.79%	4.97%
Previous percutaneous coronary intervention	1.47%	5.30%
Congestive heart failure 30 days before surgery	1.28%	5.48%
Current pneumonia	0.91%	5.86%
CVA/Stroke with neurological deficit	0.9%	5.86%
History of revascularization/amputation for peripheral vascular disease	0.87%	5.90%
History of myocardial infarction 6 months before surgery	0.85%	5.91%
CVA/Stroke with no neurological deficit	0.66%	6.10%
History of transient ischemic attacks	0.58%	6.18%
History of angina 1 month before surgery	0.53%	6.24%

Abbreviations: COPD, chronic obstructive pulmonary disease; CVA, cerebrovascular accident; mFI, modified frailty index.

**Table 9:** Descriptive statistics of individual modified frailty index variables (mFI) in the frailest population (patients with mFI ≥ 3) that experienced mortality

mFI variable	Mortality	
	Yes (%)	No (%)
Hypertension requiring medication	23.36%	3.58%
Functional health status prior to surgery	22.22%	4.72%
Diabetes	10.18%	16.76%
History of severe COPD	8.57%	18.37%
Impaired sensorium	8.56%	18.38%
Previous cardiac surgery	7.09%	19.85%
Congestive heart failure 30 days before surgery	5.52%	21.42%
Current pneumonia	4.86%	22.08%
Previous percutaneous coronary intervention	4.71%	22.23%
CVA/Stroke with neurological deficit	3.43%	23.51%
History of myocardial infarction 6 months before surgery	3.29%	23.65%
History of revascularization/amputation for peripheral vascular disease	3.18%	23.76%
CVA/Stroke with no neurological deficit	2.49%	24.45%
History of transient ischemic attacks	2.37%	24.57%
History of angina 1 month before surgery	1.47%	25.47%

Abbreviations: COPD, chronic obstructive pulmonary disease; CVA, cerebrovascular accident; mFI, modified frailty index.

## Discussion

We have shown that the mFI is a valid tool for the preoperative risk assessment of several cardiac and respiratory postoperative complications, and mortality. We have also analyzed the major determinants of frailty in a cohort of the frailest patients from this study population. Our study uniquely (1) investigates the association of frailty as determined by the mFI with specific postoperative cardiac complications (myocardial infarction and cardiac arrest), postoperative respiratory complications (unplanned reintubation, pneumonia and prolonged ventilation), and mortality in the EGS population; and (2) evaluates the individual variables comprising the mFI score to assess their distribution in the frailest patient subgroup that experienced these complications or mortality. By grouping the frailest patients and then identifying the frequency of each mFI variable by adverse outcome, it was possible to see which variables played the most significant role in the morbidity and mortality of this population.

Previous research on the association of frailty with postoperative cardiac and respiratory complications combined cardiopulmonary outcomes. 21 Additional studies explored the relationship between single disease states and specific cardiac and respiratory complications among patients who underwent EGS, 22 while others focused on the impact of cardiopulmonary outcomes on the EGS population as a whole, rather than the impact of these complications on frail patients. 13 Our project was, therefore, novel as it studied the relationship between frailty and specific postoperative cardiac and pulmonary outcomes in the EGS population.

We identified a significant association between the mFI and the adjusted odds of unplanned reintubation, pneumonia, and prolonged ventilation, separately. The odds of patients having unplanned reintubation, pneumonia, or prolonged ventilation were greater for all patients with an mFI score ≥ 1 compared with those with an mFI score of 0. Furthermore, the odds of each outcome increased with increasing mFI score; the only exception being a of the loss of trend when pneumonia was tested with mFI of 3 and mFI of 4. These findings correspond with previous research illustrating that among patients who underwent unplanned reintubation, older and more frail patients (as assessed by the mFI) had an increased risk of mortality. 24

We also identified a significant association between increasing mFI and the adjusted odds of cardiac arrest and myocardial infarction. The odds of myocardial infarction had the strongest association with frailty; a patient with an mFI score of 1 had 9.862 (95% CI: 7.051–13.792) the odds of myocardial infarction compared with a patient with an mFI score of 0. Similarly, patients with mFI scores ≥ 4 had 41.794 (95% CI: 29.554–59.104) times the odds of myocardial infarction compared with a patient with an mFI score of 0. This finding is of interest considering our identification of hypertension requiring medication as the most frequently present variable in patients of the frailest cohort (mFI score ≥3) that experienced cardiac complications, respiratory complications, or mortality. This finding is in agreement with a previous study by Dasgupta et al describing the association between frailty and postoperative complications in older adults with medical problems. 23 Dasgupta et al used the Edmonton Frail Scale to measure frailty in elderly patients undergoing elective, noncardiac surgery; however, they reported no specific association between patients on oral beta blockers before surgery and postoperative complications. 18 23 It is possible that the mFI is better able to assess the impact of frailty on postoperative complications in the EGS setting, revealing additional predictive factors for postoperative outcomes. This correlates with previous research that has identified the mFI as the most appropriate frailty index for calculating the risk of postoperative adverse outcomes and mortality in the EGS setting. 15 It is also possible that EGS, rather than elective noncardiac surgery in the study by Dasgupta et al, serves as a considerably more significant stressor in frail patients, involving more factors that are significantly associated with postoperative complications. 18

We also found that functional status prior to surgery was frequently correlated with cardiac and respiratory complications, and mortality. This is in line with previous studies that have identified preoperative functional health status as an important predictor of outcomes after colorectal surgery, 24 ventral hernia repair, 25 hepatic resections, 26 and infrainguinal bypass surgery. 27 It is also congruent with Scarborough et al's research that studied 30-day postoperative outcomes and found that functionally dependent patients experienced a 75% greater odds of mortality and 51% greater odds of major morbidity than functionally independent patients. 28

Risk assessment in the elderly population undergoing emergency surgical procedures has been identified as a critical component of health care. 29 Recent literature has emphasized the need for an accurate assessment of the suitability of each patient for high-intensity surgical treatment, especially given the increase in presentation of frail patients in this setting. 30 A recent systematic review concluded that frailty predicts postoperative mortality, complications and prolonged length of stay, and identified frailty assessment as a valuable tool in perioperative assessment. 31 Correctly identifying frailty can guide decision making and aid in the informed consent process involving patients and families. 32 We have shown, along with previous studies, 21 that the mFI is a valid predictor of morbidity and mortality in frail patients undergoing EGS. The mFI is, therefore, a useful tool for clinical decision making in the EGS setting and may also be helpful for efficient allocation of hospital resources and opportunities for early intervention in high-risk frail patients.

### Study Limitations and Strength

The high-quality of data collection and size of the NSQIP database allowed us to identify statistically significant findings in the relationship between frailty and mFI with valuable clinical implications. Our study has shown the ability of mFI to be used as a predictor of cardiopulmonary outcomes in the EGS population. This study has several limitations, including some that are inherent to the usage of administrative data. Though NSQIP is widely used in health services research due to its rigorous data collection and identification of postoperative complications, hospitals that participate in NSQIP may not be representative of the entire breadth of US hospitals. Hospitals participating in NSQIP have differences in patient volume, practice style, and case mix compared with hospitals not in NSQIP. 33 Such differences impact the generalizability of our findings to hospitals not participating in NSQIP. These differences also help explain the discrepancy between our study's population demographics and those published by Richardson et al as detailed in the introduction. Approximately 25% of our study participants were ≥ 65 years of age, while Richardson et al found that adults ≥ 65 years accounted for ∼50% of all emergent operations. 2 This difference in population demographics may also have resulted from the retrospective nature of our study. Retrospective studies are subject to potential confounding or self-reporting biases. While our study has adjusted for additional predictors, there may be other, unknown confounders affecting our results. Self-reporting bias, though likely minimized by the collection of data by trained clinical reviewers who sample cases on an 8-day cycle, 20 may lead to a misrepresentation of the volume, type of practices, and outcomes of hospitals participating in NSQIP. This would affect the classification of frailty and the magnitude of complications in our study. Future studies should focus on the prospective validation of the mFI in the EGS population in the context of specific cardiopulmonary postoperative complications and mortality.

Lastly, in our study, all Hosmer–Lemeshow showed *p* values of < 0.001, which contradicts a good-fit model ( Table 3 ). While Hosmer–Lemeshow was used to assess goodness-of-fit, the use of the test has been debated when there are a large number of covariates in regression models or if a model has been overfit. 34 35 Therefore, this test was not used as the sole indicator for goodness-of-fit. The high values of the C-statistics for each model provided support that our models were valid ( Table 3A ). 34 Sensitivity analyses also supported the goodness-of-fit of our final models as the *p* values of each mFI category were retained following adjustments to the initial, unadjusted models ( Table 3B ). Additionally, the VIF results indicated a lack of multicollinearity in the final models ( Table 3C ). 36 Multicollinearity occurs when predictor variables are correlated with each other. 36 Inclusion of these variables together in one model creates redundancy that can mask the true effect between predictors and the outcome. 36 Since our VIF values were low (range: 1.004–2.070), we have supported that multicollinearity does not exist in our final models. Because there was evidence on multiple tests that the models found were valid, we concluded that our models were appropriate despite the Hosmer–Lemeshow test results.

## Conclusions

The mFI can successfully predict the odds of postoperative cardiopulmonary complications in the EGS population and is significantly associated with unplanned reintubation, pneumonia, prolonged ventilation, cardiac arrest, myocardial infarction, and mortality. Specific variables comprising the mFI score, including hypertension requiring medication and functional status prior to surgery, have the most significant impact on the frailest patients who experience these cardiopulmonary complications or mortality.
